# Case report: Fatal Borna virus encephalitis manifesting with basal brain and brainstem symptoms

**DOI:** 10.3389/fneur.2023.1305748

**Published:** 2024-01-25

**Authors:** Athanasios Lourbopoulos, Lea Schnurbus, Ricarda Guenther, Susanne Steinlein, Viktoria Ruf, Jochen Herms, Klaus Jahn, Volker Huge

**Affiliations:** ^1^Department of Neurology and Neurointensive Care, Schoen Clinic Bad Aibling, Bad Aibling, Germany; ^2^Institute for Stroke and Dementia Research (ISD), LMU Munich University Hospital, Munich, Germany; ^3^Center for Neuropathology and Prion Research, LMU, Munich, Germany; ^4^German Center of Vertigo and Balance Disorders (DSGZ), University of Munich (LMU), Munich, Germany; ^5^Department of Anaesthesiology, LMU Munich University Hospital, Munich, Germany

**Keywords:** Borna virus-1, fatal encephalitis, meningoencephalitis, viral infection, brainstem dysfunction, basal brain dysfunction, diagnostic algorithm

## Abstract

**Background:**

Since the first report of fatal Borna virus-1 (BoDV-1) encephalitis in 2018, cases gradually increased. There is a lack of diagnostic algorithm, and there is no effective treatment so far.

**Case presentation:**

We report an acute BoDV-1 encephalitis in a 77-year-old female with flu-like onset, rapid progression to word-finding difficulties, personality changes, global disorientation, diffuse cognitive slowness, and gait ataxia and further deterioration with fever, meningism, severe hyponatremia, epileptic seizures, cognitive decline, and focal cortical and cerebellar symptoms/signs. The extensive diagnostic workup (cerebrovascular fluid, serum, and MRI) for (meningo-)encephalitis was negative for known causes. Our empirical common antiviral, antimicrobial, and immunosuppressive treatment efforts failed. The patient fell into coma 5 days after admission, lost all brainstem reflexes on day 18, remained fully dependent on invasive mechanical ventilation thereafter and died on day 42. Brain and spinal cord autopsy confirmed an extensive, diffuse, and severe non-purulent, lymphocytic sclerosing panencephalomyelitis due to BoDV-1, affecting neocortical, subcortical, cerebellar, neurohypophysis, and spinal cord areas. Along with our case, we critically reviewed all reported BoDV-1 encephalitis cases.

**Conclusion:**

The diagnosis of acute BoDV-1 encephalitis is challenging and delayed, while it progresses to fatal. In this study, we list all tried and failed treatments so far for future reference and propose a diagnostic algorithm for prompt suspicion and diagnosis.

## Introduction

Since the first reports of fatal Borna virus-1 (BoDV-1) acute encephalitis in 2018 (1, 2), the number of such cases has gradually increased to a few per year, being endemic in regions of South Germany and Central Europe (3–5). BoDV-1 infection is a potentially lethal zoonosis, with an asymptomatic course in its natural host (the bicolored white-toothed shrew, *Crocidura leucodon*) (3, 4). Infections probably occur via the uptake of contaminated virus-containing particles via the olfactory route (3). Clinical suspicion and diagnosis remain challenging because the disease symptoms are usually diffuse, while the disease rapidly progresses to irreversible and (usually) fatal brain damage. In such cases, a negative extended diagnostic panel of common viral, autoimmune, or paraneoplastic etiologies (6–9) usually leads to the diagnosis of an “encephalitis of unknown etiology” (8). However, as the mortality of BoDV-1 encephalitis reaches 95%–100% and there is no proven effective treatment so far, any clinical sign or laboratory test that facilitates its early diagnosis is desirable to initiate early supportive or off-label treatment efforts.

Here, we report a fatal case of BoDV-1 encephalitis with rapidly progressive and severe basal brain and brainstem failure. Based on the autopsy results and a critical review of all reported cases, we suggest a potential diagnostic algorithm for prompt diagnosis.

## Case description

A 77-year-old female living in a rural area of southern Germany developed, in spring 2020, initially flu-like symptoms with nausea and vomiting lasting for 3 days (without fever or reported headache), that deteriorated to additional word-finding difficulties, gait ataxia, global disorientation, diffuse confusion, and diffuse cognitive slowness in the next 2 days. She was admitted to the hospital 5 days after symptom onset. Her previous neurological status was normal and fully active for her age. Previous medications included L-thyroxin substitution due to thyroidectomy and anti-Xa anticoagulation due to atrial fibrillation and antihypertensives. Retrospectively, only in light of the final diagnosis, the patient’s family recalled a possible wild-animal attack on the patient’s domestic chickens a few days before the onset of symptoms as a potential origin of infection.

On examination upon admission (day 0), the patient was non-febrile, disoriented with cognitive slowness, diffuse confusion with severe attention deficits and reduced alertness, reduced concentration and execution capacities, mild apraxia, mild word-finding deficits, and diffuse cerebellar symptoms (mild gait-ataxia and dysmetria, saccadic eye pursuit). Meningeal signs were negative at presentation. The rest of physical examination was unremarkable. The acute brain CT scan (with CT angiography) was normal. The chest CT scan was also unremarkable. On day 2 after admission, a contrasted MRI scan of the brain showed preexisting microangiopathic alterations, without signs of encephalitis or an acute vascular event. Hematologic testing and blood chemistry at presentation (Supplementary Table 1) revealed neutrophilic leukocytosis (up to 11,900/μL, 82% neutrophils) along with severe presenting euvolemic hyponatremia (121 mmoL/L). Renal function was normal, peripheral edemas were absent, and no evidence of poor perfusion was present. The electroencephalogram (EEG) on day 2 showed a theta-slowing without any sign of epileptic activity or triphasic waves. The initial lumbar puncture (day 2) found a clear cerebrospinal fluid (CSF) with 8 cells/μL (mononucleosis), elevated total protein (62 mg/dL), CSF lactate of 3.0 mmoL/L (range < 2.4 mmoL/L), normal glucose and signs of mild blood–brain-barrier (BBB)-leakage without intrathecal IgG production (based on the Reiber diagram), cumulatively indicative of a mild non-bacterial inflammatory process. A viral encephalitis was suspected, and a corresponding CSF- and serum-diagnostic panel was ordered (8), which was negative for common bacterial or viral encephalitis (CSF PCR for HSV1/2, VZV, CMV, EBV, and serology for neuroborreliosis and endemic febrile seasonal meningoencephalitis [FSME]) and paraneoplastic antibodies.

A therapy with acyclovir, levetiracetam, and high-dose methylprednisolone was empirically initiated until the availability of the CSF results, yet without any treatment success. Hyponatremia was slowly corrected with oral substitution and fluid restriction. On day 3, the patient further deteriorated, with fever, meningism, reduced level of consciousness (somnolence), aggravated gait-ataxia, saccadic movement disorder, deterioration of cognitive functions, epileptic seizures, global aphasia, and signs of latent right pyramidal lesion (subtle pronator drift and positive Babinski sign). Due to further development of tachypnea and respiratory instability, she was prophylactically admitted to the intensive care unit (ICU). At that stage, the clinical and laboratory data supported a rapidly progressive severe (meningo-) encephalitis of yet unknown etiology (8), that manifested as a diffuse cortical process with additional diencephalic, brainstem, and cerebellar insult.

**Figure 1:**
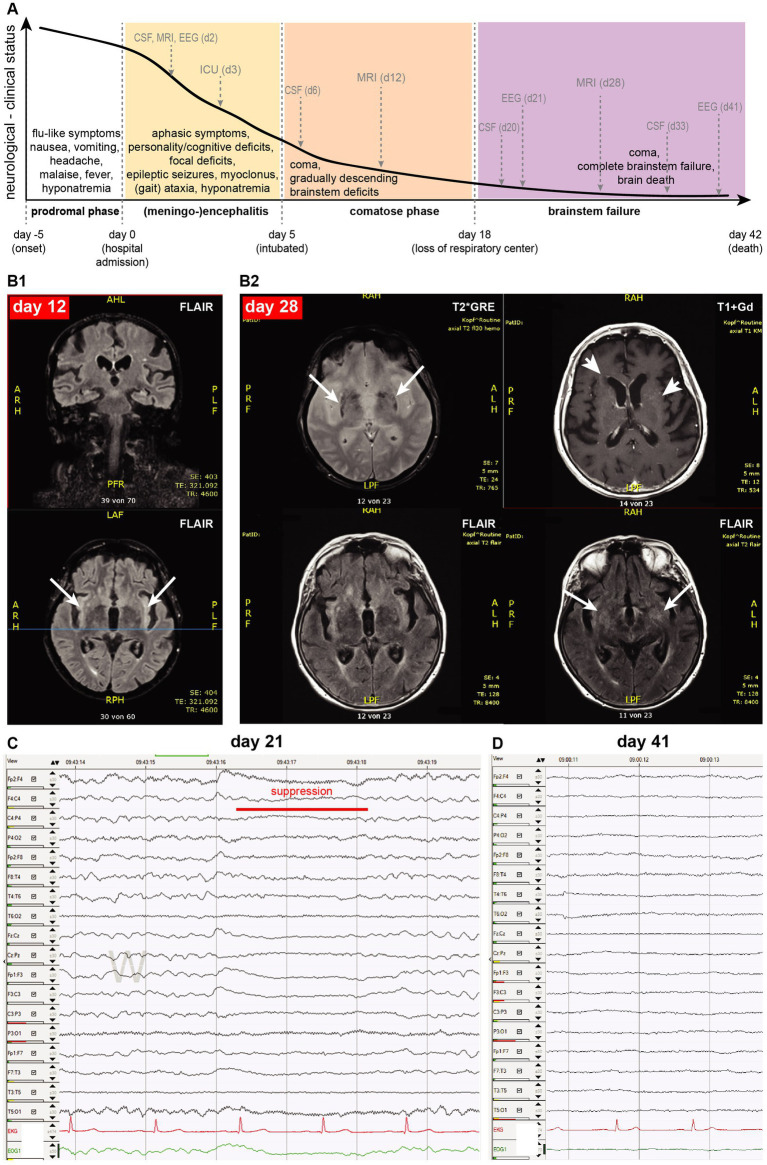
**(A)** Clinical course of the patient, shown as an arbitrary line of her neurological/clinical status decline (y-axis) against time (x-axis): grey arrows indicate running of main diagnostics as described in the text, dotted gray vertical lines indicate milestones of her disease course (onset, admission, intubation, and loss of respiratory center). **(B1)** The brain MRI on day 12 was mainly unremarkable, except for mild FLAIR signal increase in both insular cortexes (arrows). **(B2)** The MRI scan on day 28 showed age-related likely mineralization alterations in the basal ganglia (T2*GRE: arrows, present also in MRI of day 12, not shown here), diffuse and punctual gadolinium enhancement (T1 + Gd: arrowheads), and subtle expansion of increased FLAIR signal (FLAIR: arrows). **(C)** EEG on day 21 shows spontaneous burst suppression (red line) between theta-delta diffuse slowing, while the patient was clinically comatose. **(D)** On day 41, there is a complete absence of EEG-activity.

On day 5, the patient became comatose and was intubated for ventilator support. All repeatedly performed chest- and brain-CT scans remained unremarkable. The contrasted brain MRI scan on day 12 (Figure 1B1) revealed a discretely increased signal in the insula on the FLAIR sequence and discrete punctuate gadolinium enhancement in the frontal and temporal lobes without corresponding correlates in FLAIR sequences. This signal was “atypical” for other causes of viral encephalitis (10, 11). The patient remained comatose despite complete cessation of any analgosedation and was tracheotomized. Autonomic instability manifested as alternating hypertensive and hypotensive phases on a relative hypertensive background with a normal heart rate. A developed hypothermia required active warming for several days. Progressively and fastly, the patient lost cranial nerve functions and brainstem reflexes (pupillary, corneal, ciliar, and pharyngeal/laryngeal brainstem reflexes) by day 18, with complete dependence on invasive mechanical ventilation.

Her EEG continued to deteriorate, showing a generalized theta-delta rhythm with spontaneous burst suppression on day 21 (Figure 1), indicating severe encephalopathy. Due to irresponsiveness to prior treatments, a quadruple anti-tuberculosis antibiotic regime was also empirically initiated but also proved ineffective. A second round of intravenous methylprednisolone (1 g /d, for 5 days) was also ineffective.

An extensive serum- and CSF-diagnostic panel for differential diagnosis of encephalitis was repeated with additional diagnostic lumbar punctures on days 6, 20, and 33 (8, 12, 13). CSF showed a progressive lymphocytosis (increase of CSF cells at 13, 40, and 110, respectively, with >70% lymphocytes), progressive increase of total protein levels (49.7, 113, and 142 mg/dL for days 6, 20, and 33 respectively), lactate increase (3.1, 5.3, and 6.1 mmol/L respectively), increased IgG intrathecal production (53, 99, and 315 mg/dL), and constantly normal glucose. Pathologically increased CXCL13 on day 6 indicated an unspecific intrathecal B cell-related immune activation. Cumulatively, any potential local and endemic viral or bacterial causes known at that timepoint were excluded through the repeated CSF and serum diagnostic (PCR or IgM/IgG serology in serum and/or CSF). Specifically, CSF samples were PCR-negative for HSV1 and HSV2-, VZV-, CMV-, Picorna viruses (enterovirus, echo-viruses, and Coxsackie viruses), EBV-DNA and tuberculosis; serologically negative for an acute infection of rickettsies, toxoplasma, Listeria, HIV, JC-virus, rabies, HSV-6, HTLV-I/II, rotavirus, norovirus, Coxsackie, and febrile seasonal meningoencephalitis (FSME); a treponema pallidum infection was serologically (TPHA-test) excluded. SARS-CoV2 infection was repeatedly excluded via saliva and CSF PCR tests. Mumps and measles serology revealed an old infection, an acute one was excluded with PCR in CSF. Evaluation for non-infectious causes was unrevealing, including paraneoplastic markers and anti-neuronal antibodies, autoimmune etiologies, vasculitis, acute demyelinating pathology, degenerative processes (e.g., sporadic Creutzfeldt–Jacob disease), or an epileptic cause of the symptoms (e.g., a possible non-convulsive status epilepticus or epileptic discharges with postictal phenotype). NSE, oligoclonal bands, tau-protein in CSF, and b-amyloid-42/40 ratio were normal. The rare causes of severe encephalitis in Central Europe (Japanese encephalitis, dengue encephalitis, or West Nile encephalitis) were clinically, radiologically, or epidemiologically also excluded (14).

On day 27, we verified the loss of brainstem respiratory center function (apnea) in our comatose patient. A contrasted MRI brain scan performed on day 28 (Figure 1B2) did not correlate with the severity of her clinical status, showing only a subtle expansion of increased FLAIR signal compared to previous scans, some new punctual or diffuse gadolinium enhancement, and a diffuse increase of T2* signal in basal ganglia probably attributed to normal aging. Eventually, the EEG on day 41 showed a complete absence of electroencephalographic activity. A further continuation of critical care treatment was deemed to be futile, and after discussions with her family, the patient died 42 days after admission.

**Figure 2:**
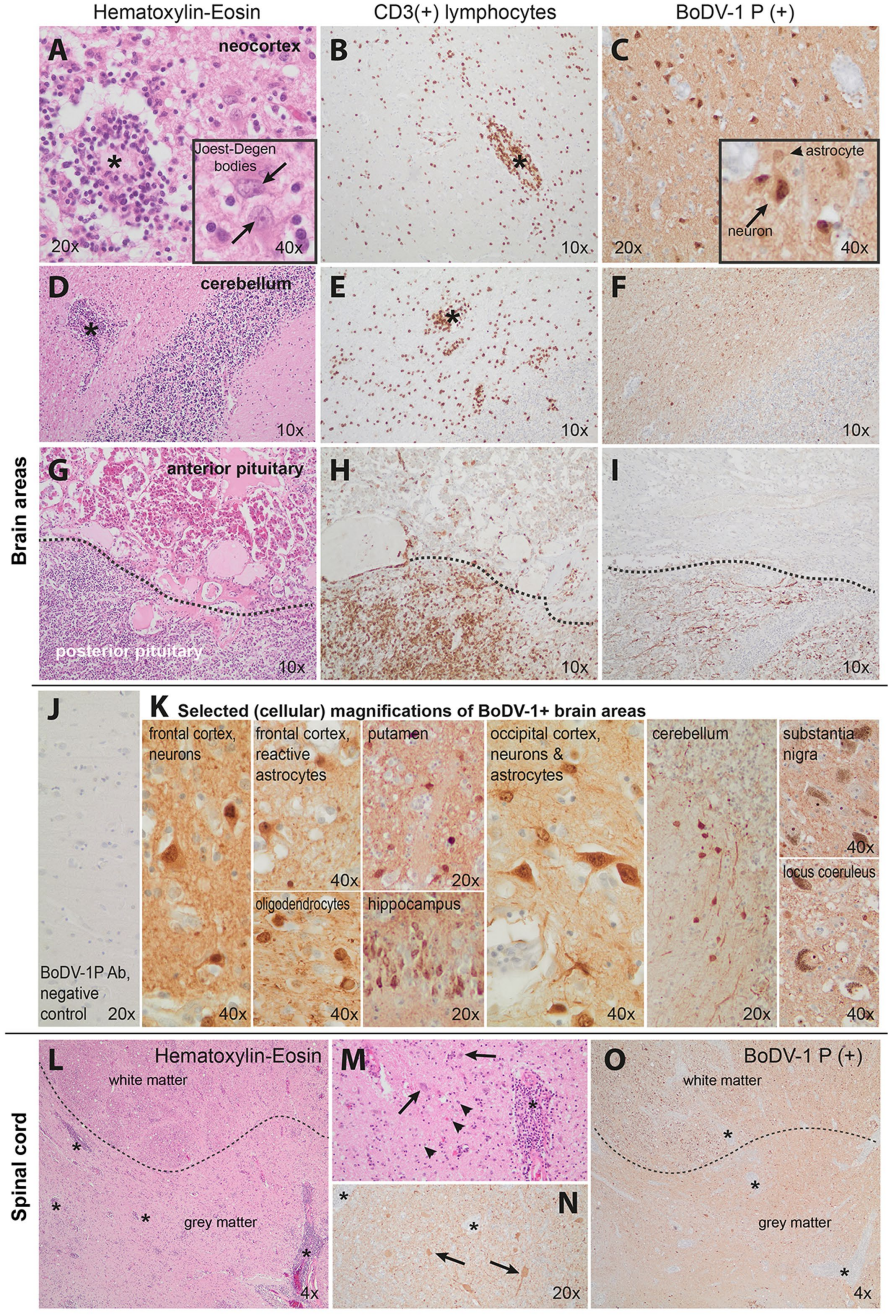
Pathology of our Borna-virus case in neocortical **(A–C)**, cerebellar **(D–F)**, pituitary **(G–I)**, and upper cervical spinal cord **(L–O)** areas. **J,K** show selected cellular magnifications of brain areas. Hematoxylin–eosin or CD3 (for T lymphocytes) and Borna-virus-type-1 immunohistochemistry are illustrated here. Prominent perivascular as well as diffuse lymphocytic infiltration was found in all neocortical areas (**A,B**, asterisks at indicative infiltrations). This was accompanied by marked reactive astrogliosis with bizarrely enlarged reactive astrocytes (indicative of sclerosing encephalomyelitis), some of them with eosinophilic intranuclear inclusions (**A**-inset, arrows point at Joest–Degen bodies, i.e., distinct eosinophilic intranuclear inclusions). Borna disease virus 1 (BoDV-1) immunohistochemistry revealed strong positivity in neurons as well as the surrounding neuropil **(C)**. Distinct inflammatory infiltration was also seen in the cerebellum (**D,E**, indicative asterisks) though reactive changes were less pronounced. The BoDV-1 signal was primarily visible in the white matter **(F)**. The neurohypophysis (**G–I**, lower half) demonstrated massive lymphocytic infiltration **(G,H)** and presence of viral BoDV-1 antigens **(I)** in contrast to a relative absence of inflammatory infiltrates in the adenohypophysis (**G–I** upper half). **J** shows the negative control (frontal cortex of a patient with progressive supranuclear palsy) of our staining. Photos in **K** demonstrate selected cellular magnifications from lesioned areas, indicating Borna virus positivity in neurons, oligodendrocytes, and astrocytes. Finally, **L–N** show severe upper spinal cord pathology as well: severe inflammation (indicative asterisks of smaller or larger inflammatory foci) affected and indistinct borders between the gray and white matter (dotted lines in **L** and **O**), neuronal loss, and strong reactive astrogliosis (arrowheads pointing at glial cells) indicative of sclerosing encephalitis; **N** and **O** show BoDV-1+ signal in the spinal cord. **M** and **N** are magnifications of hematoxylin–eosin and BoDV-1 staining, respectively. Cumulatively, the pathology indicated a non-purulent, lymphocytic sclerosing panencephalomyelitis with detection of BoDV-1-typical eosinophilic, spherical intranuclear Joest–Degen inclusion bodies in accordance with previous neuropathology studies (16).

The family provided informed consent for a diagnostic autopsy. A BoDV-1 infection with induced non-purulent, lymphocytic sclerosing panencephalomyelitis was diagnosed as the cause of the fatal encephalitis, both by routine immunohistopathology (we used an antibody against the phosphoprotein (P) antigen (4), kindly provided by Dr. D. Rubbenstroth, at a dilution of 1:3000) and real-time PCR, as previously established (4, 15). The sclerosing aspect is represented by the strong glial, primarily astrocytic activation with enlarged hypertrophic astrocytes in both brain and spinal cord (Figure 2). “Joest–Degen bodies” [i.e., distinct eosinophilic intranuclear inclusions, considered typical in BoDV-1 infection (15)] were also found in brain and spinal cord (Figure 2). The sequence analysis of the virus genome, using methodology previously established (15, 17), identified it within cluster 1A, phylogenetic similar to those isolated from the southeast area of Bavaria in other cases. BoDV-1 antigens and severe related pathology were detected in multiple areas in the brain, such as neocortical (e.g., frontal and occipital cortexes), hippocampus, subcortical (putamen and basal ganglia), cerebellar (nuclei and cortex), brainstem nuclei (e.g., pons nuclei, substantia nigra, and locus coeruleus) and neurohypophysial areas, as well upper cervical spinal cord (see Figure 2), explaining the clinical presentation of the patient.

## Discussion

We report a fatal case of BoDv-1 encephalitis in a 77-year-old habitant of Southern Germany, presented as rapidly progressive diencephalic and brainstem failure, leading to brain death. After reviewing all reported cases so far (Table 1), we propose a potential diagnostic algorithm to narrow down the differential diagnosis.

**Table 1:** Reported cases of Borna virus encephalitis since the first report in 2018 (18) and up to November 2023.

No of cases, year of onset (Ref.)	Fatality (time to death)	Symptoms, signs; reported exposure to wild animals (yes/no)	Used medications	CSF WBC (cells/μL)	MRI findings (normal/abnormal and localization)	Reported abnormalities in pathology (biopsy, autopsy, or both)
2, 2023 (17)	2/2 (4w, previously reported in Eisermann et al. (19); 10w)	Fever, headache, severe malaise, epileptic seizure (case 1); fever, headache, vomiting, disorientation (case 2) Contact to animals: yes (2/2)	Case 1: Acyclovir, doxycycline, steroids, IVIG, ribavirin, favipiravir (➔ death). Case 2: favipiravir, ribavirin, remdesivir, steroids, cyclosporin, MMF, some given intrathecal (➔ transient improvement, eventually death)	Case 1: ND Case 2: reported “mono-lymphocytosis” (118/μL)	Abnormal. Case 1: Cortical (parietal), subcortical (basal ganglia) increased FLAIR-signal and cytotoxic edema; Case 2: Cortical (temporal, temporomesial), subcortical (basal ganglia) and brainstem increased FLAIR-signal, later brain atrophy	Case 1 (biopsy): sclerosing lymphocytic encephalitis, neuronal loss and Joest–Degen bodies. Case 2 (autopsy): sclerosing lymphocytic panencephalitis, BoDV-1 in “all brain regions” (e.g., in limbic system, olfactory structures, thalamus, frontotemporal/ parietal areas basal ganglia, and brainstem).
1, 2022 (20)	1/1 (7w)	Flu-like symptoms, progressive confusion and speech disorders, coma. Contact to animals: ND	Steroids, PE (➔ death).	ND	Abnormal. Cortical (insular), subcortical (basal ganglia) and hippocampal increased FLAIR-signal, subinsular hemorrhage	Autopsy: performed but no clear BoDV-1 related-data reported. Biopsy (caudate): sclerosing lymphocytic encephalitis, Joest-Degen bodies.
2, 2022 (21)	2/2 (10w, 16d)	Fever, flu-like symptoms, headaches, dysphagia, vigilance decline, epileptic seizures, temperature regulation disorders, dyspnea, loss of brainstem reflexes, coma. Contact to animal: in 1/2 of cases	Case 1: steroids, PE, antiepileptics, Rituximab, Cyclophosphamid, favipiravir (➔ transient stabilization, eventually death) Case 2: antiepileptics, steroids, IVIG, PE, Cyclophosphamid, rituximab (➔ death)	Case 1: reported “lymphocytosis” (23/μL) Case 2: reported “pleocytosis” (49/μL)	Abnormal. Case 1: Cortical (frontoparietal, insula), subcortical (basal ganglia) and hippocampal increased FLAIR-signal; Case 2: Hippocampal increased FLAIR-signal.	Case 1 (biopsy): BoDV-1+, lymphocytic encephalitis. Case 2 (biopsy): BoDV-1+
1, 2022 (22)	1/1 (30d)	Confusion, dizziness, vomiting, memory impairment, BBB disruption, respiratory deterioration, coma, and brainstem involvement.	ND (➔ death)	Reported “leukocytosis” (41/μL)	Normal.	Autopsy: BoDV-1+ in frontal cortex, optic, and peripheral nerves
3, 2022 (18)	2/3 (5w; unknown)	Case 1: dysphasia, vigilance decline, epileptic seizures, sopor and ocular bulbus divergence. Patient reported as “alive.” Cases 2: Fever, dysphasia, ataxia and progressive vigilance decline, epileptic seizures, loss of brainstem reflexes, and coma (terminal). Case 3: ND. Contact to animals: living in rural areas but ND of animal contact	Case 1: ND (➔ alive) Case 2: ND (➔ death) Case 3: ND (➔ death)	ND	Abnormal. Case 1: Cortical (temporomesial, insula), subcortical (basal ganglia, thalami) “widespread” increased FLAIR-signal, diffuse edema; Case 2: “widespread” increased FLAIR signal, especially of thalami (subcortical). Case 3: ND	Case 1: no autopsy or biopsy. Case 2 (autopsy): BoDV-1+ in all brain areas examined (frontal, parietal, occipital lobes, hippocampi, thalami, basal ganglia, brainstem, and cerebellum) Case 3 (“histology”): ND
2, 2021 (23)	2/2 (5 m; 3 m)	Cognitive deterioration, apathy, brainstem involvement, respiratory deterioration, coma. Contact to animals: ND	ND for both cases (➔ death)	ND	ND	Case 1: no autopsy or biopsy Case 2 (meningeal biopsy): BoDV-1+
1, 2021 (24)	1/1 (4w)	nausea, psychomotor slowing, apathy, temporary sensory aphasia, ataxia and dyspnea, paraplegia, and coma. Contact to animals: yes	Antibiotics, antimycotics, acyclovir, steroids (➔ death)	Reported “lympho-monocytic pleocytosis” (109/μL)	Normal	Autopsy (death in 2017, re-autopsy in 2020): non-purulent meningoencephalitis, BoDV-1+ in all brain areas (various cerebrocortical areas, basal ganglia, hippocampi, cerebellum, and brainstem)
3, 2019–2020 (19)	3/3 (3-4w in all cases) One of these cases is also reported in Liesche et al. (15) and Niller et al. (25)	Fever, headache, encephalitis, coma/epileptic seizures/confusion. Contact to animals: living in rural areas with animal contact	Case 1: antibiotics, acyclovir, steroids, IA (➔ death) Case 2 and 3: ND (➔ death)	Case 1: 180 cells/μL (retrieved data from Niller et al. (25), casepatient 8). Cases 2 and 3: ND	Case 1: Normal Cases 2 and 3: ND	Case 1 (autopsy): BoDV-1+ (retrieved data from Liesche et al. (15), case patient 6, show mainly brain stem and subcortical nuclei lesions). Case 2 and 3: no autopsy.
19, <2020 (14)	(19/19) 9 out of those 19 cases were previously included in other case studies (mortality 38 ± 22d)	MRI study. Fever or flu-like episodes, focal neurological symptoms, progressive encephalopathy, dysarthria, visual hallucinations. Contact to animals: ND	Data reported in other publications for some of the cases, see (2, 15, 26).	Reported “pleocytosis” (no cells/μL are reported)	MRI Study of all cases: On day 1: 53% MRIs with lesions. On day 26 ± 13: most-affected area the head of caudate nucleus, followed by hippocampus, insula, parahippocampal gyrus, temporal pole, thalamus, frontal pole, putamen, striatum, gyrus rectus operculum.	Autopsy in a representative patient included in Liesche et al. (15).
8, 1999–2019 (25)	8/8 (1 co-reported in Eisermann et al. (19): death within 16-57d after admission in all cases)	Headache, fever, confusion, ataxia, progressive confusion, epileptic seizures, focal deficits, coma, brainstem deficits/death. Contact to animals: yes for 6/8, ND for 2/8.	Combinations of antibiotics, acyclovir, ganciclovir, steroids, cidofovir, cyclophosphamide, PE (➔ death)	Reported “leucocytosis” (19-633 μL)	Abnormal initially in 2/8 (up to day 9): cortical (temporal, frontal). Abnormal in 6/8 patients at later timepoints: cortical (frontal, temporal, peri-insular), subcortical (basal ganglia), brainstem.	Autopsies in all cases: Panencephalitis (5/8) or meningoencephalitis (2/8), accompanied by hypophysitis (1/8) and myelitis (3 in 3 patients for spinal cord was examined) or brainstem-accentuated meningoencephalomyelitis (1/8).
6, 2019 (15)	6/6 (autopsies, 1 co-reported in Eisermann et al. (19): death within 2-14w)	Flu-like symptoms, headache/fever, GBS (1 case), hemiparesis (1 case) – > focal deficits, epileptic seizure, confusion – > coma, brainstem deficits. Contact to animals: ND for 5/6; for case referred in Eisermann et al. (19) see above.	Combinations of antibiotics, PE, steroids, IVIG, acyclovir, ganciclovir, IA (➔ death)	ND	Abnormal. Variable MRI findings, refer to Table 1 in Liesche et al. (15) for each one of the 6 cases and Finck et al. (14) for collective data.	Autopsies in all cases: non-purulent panencephalomyelitis with Joest-Degen bodies, astrogliosis and variable neuronal loss. BoDV-1+ in all cases in cortical and subcortical areas, brainstem, cerebellum and spinal cord.
3, 2018 (2)	2/3 (transplant donors: 96 and 99d post-onset)	progressive ascending flaccid, sensorimotor tetraparesis with encephalitis progressing to irreversible coma and death (Cases 1 and 2) Facial palsy, anomia, cognitive deficits, optic neuritis (case 3, reported as “alive”). Contact to animals: ND	Various agents of immunosuppression (ATG, IVIG, everolimus/ tacrolimus, TPE, cyclosporine, steroids, MMF, eculizumab), ribavirin.	Case 1 and 2: “normal” Case 3: 15 cells/μL	Abnormal. Case 1: diffuse cerebral atrophy. Case 2: diffuse cerebral atrophy and reported “diffuse supratentorial and infratentorial encephalitis in FLAIR.” Case 3: micronagiopathy	Case 1 (biopsy): BoDV detection (no other reported data) Case 2 (autopsy): BoDV-1+ in brain tissue, herniation, severe non-purulent pan-meningo-encephalomyelitis and neuritis of sciatic and vagus. Case 3: no autopsy/ biopsy
1, 2018 (1)	1/1 (1 m)	Fever, headache, confusion, myoclonus, gait instability, continuous fever, brainstem deterioration. Contact to animals: yes	Antibiotics, acyclovir, steroids, PE (➔ death)	Reported “lymphocytic pleocytosis” (7/μL on day 1, 68/μL on day 7 with 90% lymphocytes)	Abnormal. Meningitis, FLAIR+ hippocampal and subcortical signal (thalami and caput nuclei caudati) brain edema on day 20, herniations	Autopsy: inflammatory lymphocytic necrotizing encephalitis with Joest–Degen inclusion bodies, astrogliosis.
1, 2019 (26)	1/1 (5 m)	GBS polyradiculitis with cytoalbuminic dissociation, EEG pathologic. Fever, coma within 14ds. Contact to animals: ND	PE, IVIG, acyclovir, antibiotics, amantadine (➔ death)	10cells/μL on day 1	Normal on admission. No more data reported	Brain biopsy (frontal): disseminated lymphomonocytic meningoencephalitis, BoDV-1+

The first column shows the number of cases, their year of disease onset, and the relevant reference; the second column shows the fatality (e.g., 2 out of 2) and, in brackets, the disease duration from symptom onset to death (time to death) for each case (w: weeks, d: days, m: months). The fourth column (used medications) shows the respective treatment efforts in each case or case series. BBB = blood–brain barrier, ND = no data reported or not done. IVIG = intravenous immunoglobulins, ATG = anti-thymocyte globulins, TPE = total plasma exchange, IA = immuneabsorption, MMF = mycophenolate-mofetinil.

BoDV-1 is a neurotropic RNA virus in birds and mammals, causing acute, subacute, or chronic persistent CNS infections (27). Our current knowledge on BoDV-1 mainly comes from *in vitro* (multiple primary cells) and *in vivo* (rats, gerbils, rhesus macaques) models, as well as studies in naturally occurring animal or human infections. The only definite currently known natural host of BoDV-1 is the bicolored white-toothed shrew (*Crocidura leucodon*), which develops a chronic persistent infection without or with mild symptoms and excretes the virus via urine and feces (16, 28, 29). The virus is reported to cause encephalitis with movement and behavioral disorders in animals (e.g., horses, sheep, and cats), chronic infections in various species (27, 30), or fatal spillover infections in horses and sheep (3, 4). In humans, BoDV-1 infection is at present endemic in South Germany and Central Europe, in regions close to nature (in a stand-alone location or on the fringe of the settlement) presumably due to higher exposure risk to infected animals (5). Despite previous discrepancies regarding its transmission or disease severity in humans, it is now certain—since the first documented cases in 2018 (1, 2)—that BoDV-1 can cause acute fatal encephalitis in both immune-compromised and healthy humans. Previous single reports on a theoretical BoDV-1 involvement for psychiatric symptoms in non-symptomatic carriers (31, 32) should be interpreted very cautiously (30) as they were not independently validated, failed in interlaboratory comparisons, or could be the result of a laboratory contamination (28, 33). According to *in vivo* experimental data (rat) (3), the encephalitis probably occurs via the uptake of contaminated virus-containing particles via the olfactory route (3, 34), e.g., via contaminated dust, then transported intra-axonal and replicates from neuron to neuron (34) with subsequent expansion in astrocytes (35). Furthermore, *in vivo* experimental data in rats also indicate a monoamine nuclei and circuit viral distribution [for a detailed review see (27)], which also resembles our pathology findings (Figure 2K). A recent (2022) *in vivo* non-human primate model also supports that a non-CNS peripheral route exposure seems unlikely to be highly effective for fatal BoDV-1 infection, whereas intracerebral inoculation induces a human-like fatal acute encephalitis within a few weeks in 100% of animals (36). However, it is still not clear why the virus causes the fatal disease. The initially established *in vitro* models of BoDV-1 infection (in primary neurons and other cell lines) indicated that the virus is no-lytic (37, 38), supported also by experimental *in vivo* studies (39). On the other hand, recent *in vitro* modelling in primary neurons indicates that BoDV-1 dampens neuronal activity, suggesting a newly discovered direct neuropathogenic effect (40). *In vivo*, the virus seems to induce an increasing pro-inflammatory immune activation during BoDV-1 encephalitis (35, 39), either as part of the host’s normal immune reaction or as a dysbalanced pro-inflammatory state, with lymphocytic-mediated (preferably CD8+) degeneration of the brain (41). This suggests that the immune system may be partially responsible for the cellular loss. However, a cyclosporin-induced humoral and brain immunosuppression in a gerbil model did not inhibit the fatal encephalitis, as also shown by reported cases that used broad immunosuppression (see Table 1) but indicated a probable combined viral- and cytokine (IL-1b)-related fatal process (42). On the other hand, an experimental blockage of lymphocytic entry in the infected CNS showed some slowed disease progression in rats (43) that warrants further research.

**Figure 3:**
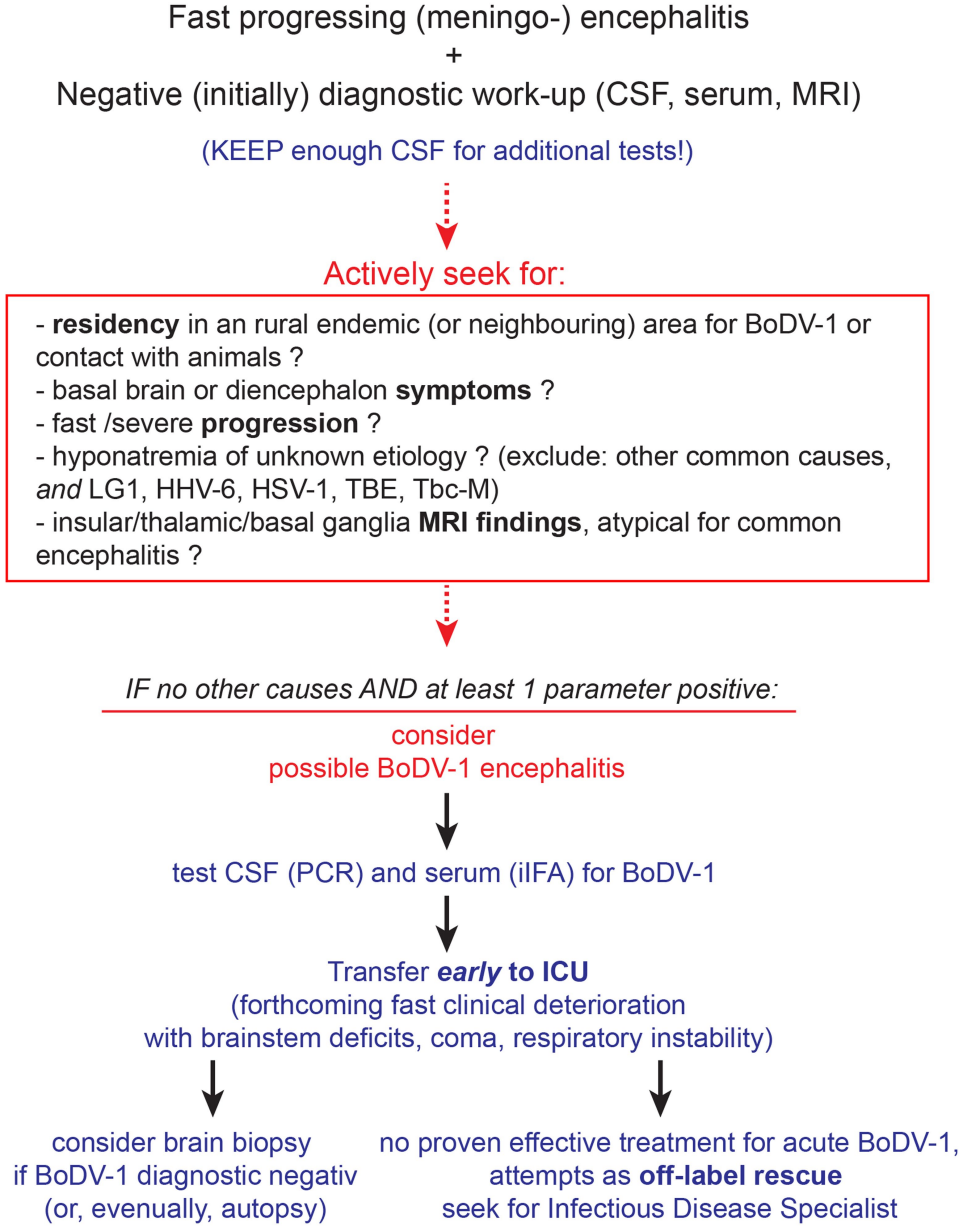
Diagnostic evaluation for BoDV-1 encephalitis. Although an empirical antiviral treatment should be initiated to cover for common causes of encephalitis until diagnostic results, it is ineffective for BoDV-1 (see Discussion and Table 1). Initial diagnostic workup should preferably be performed as fast as possible, according to guidelines, keeping the cerebrospinal fluid (CSF) sample for additional diagnostic. For CSF and serum tests, the PCR (44) and indirect immunofluorescence assay (iIFA) (45) for BoDV-1, respectively, are reported as the most sensitive and specific. Brain biopsy (and eventually autopsy) should be performed to acquire important information on this emerging disease. Abbreviations: LG1, limbic anti-LG1 encephalitis; HHV-6, human herpesvirus 6; HSV-1, herpes simplex virus type 1; TBE, tick-borne encephalitis; Tbc-M, tuberculous meningitis; ICU, intensive care unit; iIFA, indirect immunofluorescence assay.

A diagnosis of acute BoDV-1 encephalitis can now be defined based on recently proposed serological criteria (19). Along with that, we now propose a diagnostic algorithm that may increase the suspicion of the disease (Figure 3). A recently (2022) available ELISA assay can be used for serological screening (22). The PCR detection of BoDV-1 in CSF (44) and indirect immunofluorescence assay (iIFA) (45) in serum are considered currently the most reliable for BoDV-1 detection. Overall, 41 BoDV-1 cases have been reported so far (Table 1, up to November 2023), all of them in Central Europe. A cohort of MRI cases includes 19 patients, 9 out of which were reported elsewhere (14). Two cohorts with autopsy data report six (15) and eight (25) cases, one of which was included in a previous report (19). Our case is newly reported (“case 42”) and not included in any previous cohort. Cumulatively, the previously reported cases point toward T2 or FLAIR hyperintensities (Table 1) in 86% (32 out of the 37 reported data), usually located at the deep or cortical gray matter structures and nuclei of the brain (head of the caudate nucleus, thalamus, hippocampus, insula, cortical limbic system, and brainstem), with unaffected occipital lobes and cerebellar hemispheres (14, 20); normal MRI findings can be found in 13% (5 of 37 reported data, Table 1). In our patient, repeated MRI brain scans remarkably did not reflect the severity of brain pathology, even at later time points (“MRI-clinical dissociation”). On the contrary, EEG reflected the clinical severity, with spontaneous burst suppression (day 21) and eventual complete absence of electroencephalographic activity (day 41). In light of the final autopsy result, the disease course fitted to a diffuse rapidly progressive cortical, subcortical, and infratentorial involvement, spread on the diencephalon (thalamus, hypothalamus, and neurohypophysis), brainstem, and even spinal cord.

Retrospectively seen, we would point out the following. The disease began as a non-specific viral infection and evolved rapidly into a severe “encephalitis of unknown etiology.” An acute “encephalitis of unknown etiology” may account for as much as 37% of all encephalitis cases admitted to a hospital, 9% of which are fatal (6). MRI findings could only exclude other known causes of viral encephalitis (10). The progression to diffuse diencephalic and brainstem failure with eventual brain death was remarkably fast and resistant to empirical treatments, even for “encephalitis of unknown etiology.” The presenting severe hyponatremia, when not adequately explained otherwise (46, 47) could imply a few causes of viral encephalitis [limbic anti-LG1 encephalitis (48), human herpesvirus 6 (HHV-6) (49), herpes simplex virus type 1 (HSV-1) (50), tick-borne encephalitis (TBE) (51)], as well tuberculous meningitis (52), all of which were excluded by our diagnostic procedures. CSF diagnostics pointed to an unspecific intrathecal B cell-related immune activation, with relatively few lymphocytes that increased only slowly. This reflected the pro-inflammatory, lymphocytic-mediated, brain degenerative, immune activation under BoDV-1, revealed by the autopsy findings and lesion localization, that clinicopathologically explained the clinical symptoms and signs of the patient. Taken all these together, an early suspicion of BoDV-1 (to run specific diagnostic tests early) could probably be triggered by the severity, speed, and “unusual” findings of the disease (Figure 3).

There is no proven effective therapy for acute BoDV-1 encephalitis and the disease progresses fatal. All approaches remain ineffective, experimental, and as single-case efforts, based on various hypotheses. An older report of “anti-viral” efficacy of amantadine in psychiatric patients with subclinical BoDV-1 infection (53) should be critically questioned for the reasons discussed above (28, 33). Single (*in vitro* or *in vivo*) experimental studies testing antiviral compounds [ribavirin (54, 55), favipiravir (T-705) (56), amantadine (57)], cocktails of small-interfering RNAs (siRNAs) (58) or combinations of those, have all proven clinically ineffective (2, 20, 21). Alternatively, as BoDV-1 induces a severe pro-inflammatory state (15), a combination of antiviral and anti-inflammatory treatment was speculated to be beneficial. However, this approach of BoDV-1 treatment is proven so far ineffective, both preclinically *in vivo* (e.g., test of cyclosporin A) (42) and clinically in the relevant reported cases where immunosuppression was used (see Table 1 and our reported case here). A single recent report of temporary clinical and viral improvement in a 6-year-old boy, after early (at day 12 post-disease-onset) administration of favipiravir (i.v.), ribavirin (i.v. and intrathecal), remdesivir (i.v.), and triple aggressive immunosuppression should also be considered with caution as the patient eventually also died (17). The fact that two patients out of the 42 BoDV-1 encephalitis were reported alive at the time of relevant publication (see Table 1, both diagnosed via iIFA) (2, 18) is an enigma as their treatments or disease characteristics did not substantially differ from the other cases. Eventually, seen collectively, the acute BoDV-1 encephalitis progresses to death, without any effective treatment up to date.

In conclusion, diagnosis of BoDV-1 encephalitis is difficult. It requires a high degree of suspicion in fast and unusual evolving severe “encephalitis of unknown etiology” as we propose in our algorithm. Serum and CSF BoDV-1 diagnostic panels are recently available. Treatment efforts are all off-label, not evidence-based, individual trials in an otherwise fatal disease.

## Data availability statement

The datasets presented in this article are not readily available because of ethical and privacy restrictions. Requests to access the datasets should be directed to the corresponding author.

## Ethics statement

Ethical approval was not required for the study involving humans in accordance with the local legislation and institutional requirements. Written informed consent to participate in this study was not required from the participants or the participants’ legal guardians/next of kin in accordance with the national legislation and the institutional requirements. Written informed consent was obtained from the individual(s) for the publication of any potentially identifiable images or data included in this article.

## Author contributions

AL: Conceptualization, Data curation, Formal analysis, Investigation, Methodology, Project administration, Writing – original draft. LS: Data curation, Formal analysis, Writing – original draft. RG: Resources, Validation, Writing – review & editing. SS: Resources, Validation, Writing – review & editing. VR: Data curation, Writing – original draft, Investigation. JH: Supervision, Validation, Writing – review & editing. KJ: Supervision, Validation, Writing – review & editing. VH: Supervision, Validation, Writing – review & editing, Resources.
